# Effects of Hemodynamic Response Function Selection on Rat fMRI Statistical Analyses

**DOI:** 10.3389/fnins.2019.00400

**Published:** 2019-04-30

**Authors:** Shin-Lei Peng, Chun-Ming Chen, Chen-You Huang, Cheng-Ting Shih, Chiun-Wei Huang, Shao-Chieh Chiu, Wu-Chung Shen

**Affiliations:** ^1^Department of Biomedical Imaging and Radiological Science, China Medical University, Taichung, Taiwan; ^2^Department of Radiology, China Medical University Hospital, Taichung, Taiwan; ^3^Department of Medical Imaging and Radiological Sciences, Chung Shan Medical University, Taichung, Taiwan; ^4^Center for Advanced Molecular Imaging and Translation, Chang Gung Memorial Hospital, Taoyuan City, Taiwan

**Keywords:** blood-oxygen-level dependent (BOLD), electric stimulation, barrel, forepaw, boxcar function

## Abstract

The selection of the appropriate hemodynamic response function (HRF) for signal modeling in functional magnetic resonance imaging (fMRI) is important. Although the use of the boxcar-shaped hemodynamic response function (BHRF) and canonical hemodynamic response (CHRF) has gained increasing popularity in rodent fMRI studies, whether the selected HRF affects the results of rodent fMRI has not been fully elucidated. Here we investigated the signal change and *t*-statistic sensitivities of BHRF, CHRF, and impulse response function (IRF). The effect of HRF selection on different tasks was analyzed by using data collected from two groups of rats receiving either 3 mA whisker pad or 3 mA forepaw electrical stimulations (*n* = 10 for each group). Under whisker pad stimulation with large blood-oxygen-level dependent (BOLD) signal change (4.31 ± 0.42%), BHRF significantly underestimated signal changes (*P* < 0.001) and *t*-statistics (*P* < 0.001) compared with CHRF or IRF. CHRF and IRF did not provide significantly different *t*-statistics (*P* > 0.05). Under forepaw stimulation with small BOLD signal change (1.71 ± 0.34%), different HRFs provided insignificantly different *t*-statistics (*P* > 0.05). Therefore, the selected HRF can influence data analysis in rodent fMRI experiments with large BOLD responses but not in those with small BOLD responses.

## Introduction

Functional magnetic resonance imaging (fMRI) was originally introduced in 1990. Since then, it has been modified to enable investigations on different functional aspects of the brain. The most popular fMRI technique is blood-oxygen-level dependent (BOLD) contrast, which relies on local deoxyhemoglobin changes (Ogawa et al., 1990, 1992). Owing to its advantages of absent radiation burden and non-invasiveness, BOLD fMRI has become a pivotal method for understanding brain function and physiological conditions (Tsurugizawa et al., 2010; Rana et al., 2013; Wu et al., 2014; Nasrallah et al., 2015; Golestani et al., 2016). The applications of BOLD fMRI in animals such as rats, have recently received increased attention. The majority of rodent fMRI studies have been conducted by using electric stimulation to induce somatosensory stimulation and to estimate activations in the primary sensory cortex (Silva et al., 1999; Keilholz et al., 2004; Tenney et al., 2004; Weber et al., 2006; Masamoto et al., 2007; Pelled et al., 2007; Shih et al., 2009, 2011, 2013;Ramos-Cabrer et al., 2010; Just et al., 2013; Sanganahalli et al., 2013; Nasrallah et al., 2015). Previous works have emphasized the importance of rodent fMRI studies in elucidating crucial topics in neuroscience research. These topics include functional recovery (Pelled et al., 2007; Ramos-Cabrer et al., 2010), pain processing (Shih et al., 2009, 2011), and neurodegenerative diseases (Tenney et al., 2004; Sanganahalli et al., 2013).

The construction of the hemodynamic response function (HRF) of the signal response to an external stimulus is the essential step in the statistical analysis of fMRI data for identifying activation regions. The typical HRF used in rat fMRI studies is the boxcar-shape HRF (BHRF) (Keilholz et al., 2004; Pelled et al., 2007; Shih et al., 2009, 2011; Yang et al., 2013; Nasrallah et al., 2015), which is based on the standard on/off format of the external stimulation. Meanwhile, the use of the so-called canonical HRF (CHRF), a sophisticated HRF based the convolution of a BHRF with the sum of two gamma functions, has also been suggested by other groups (Kim et al., 2007; Yu et al., 2012). The advantage of BHRF is that BHRF can be used for the rapid assessment of brain activation to further refine the CHRF. Although HRF selection in human fMRI studies have been widely discussed (Aguirre et al., 1998; Handwerker et al., 2004; Shan et al., 2014), the body of research that compares the HRF selection in rodent fMRI studies remains insufficient (Chavarrias et al., 2016).

Therefore, the central objective of this study is to systematically investigate whether fMRI activation detection can be affected by the selected HRF. To achieve this objective, three HRFs (Lu et al., 2005; Lindquist et al., 2009; Nasrallah et al., 2015) were employed to model the BOLD signal. The extent of brain activation by electric stimulation is task-dependent, with whisker pad stimulation projecting larger somatosensory regions than forepaw stimulation (Yu et al., 2010). Thus, to map different sensory processing in the brain cortex, we subjected two groups of rats to whisker pad and forepaw electric stimulations. The estimated BOLD signal changes and *t*-statistics among three HRFs were compared. Such a comparison may provide recommendations for future rat fMRI studies.

## Materials and Methods

### Functional Magnetic Resonance Imaging Experiments

A total of 20 male Sprague-Dawley rats (280–345 g) were used in this study. Laboratory animals were housed in plastic cages with soft bedding and were maintained on a 12-h light/dark cycle. Food and water were available *ad libitum*, and the room was temperature controlled. This study was carried out in accordance with the recommendations of National Institutes of Health guide for the care and use of Laboratory animals. The protocol was approved by the China Medical University.

All MRI experiments were conducted on a 7T animal MRI scanner (Bruker ClinScan 70/30, Germany) with a gradient strength of 630 mT/m. A volume coil and a surface coil were used for signal excitation and reception, respectively. All rats were initially anesthetized with 4% isoflurane (ISO), and then was reduced to 1–1.2% ISO during fMRI (Liu et al., 2004). Each rat was secured in a head holder with ear bars and a bite bar to prohibit head motion. The rats were placed on a heated water pad to maintain body temperature at ∼37°C while in the magnet.

Rats were subsequently divided into two groups. In the first group (*n* = 10), needle electrodes were inserted under the skin of the left whisker pad for mapping the primary somatosensory cortex barrel field (S1BF). In the second group (*n* = 10), needle electrodes were inserted under the skin of the left forepaw for mapping the primary somatosensory cortex forelimb region (S1FL). Electric stimulation was performed by a stimulator (Isolated Pulse Stimulator Model 2100, Washington, DC, United States) supplying 3 mA, 330 μs pulses repeated at 3 Hz to either the left whisker pad or the forepaw upon demand. The stimulation paradigm of the fMRI experiment consisted of a block design. The stimulation paradigm including an initial 75 s period of resting followed by five cycles alternating 15 s of electric stimulation with 75 s of resting was implemented, with a total duration of 525 s. BOLD MR images were simultaneously acquired during this period. The BOLD imaging parameters were field of view (FOV) = 30 mm × 30 mm, matrix size = 64 × 64, 7 coronal slices, thickness = 1 mm, no gap, repetition time (TR)/echo time (TE) = 1000 ms/25 ms, and single-shot gradient echo echo-planar imaging. Anatomical images were obtained by turbo-spin-echo with scanning parameters of TR = 2560 ms, TE = 38 ms, echo train length = 7, number of excitation = 1, FOV = 30 mm × 30 mm, matrix size = 320 × 320, and slice thickness = 1 mm.

### Data Analysis

The data analysis for each animal was performed using first-level analyses in SPM8. The voxel-by-voxel statistical analysis of fMRI data was based on the general linear model (GLM) analysis. The dependent variable was the BOLD signal, and the first regressor was the HRF. Three different types of HRFs were employed in this study to test the influence of HRF selection on fMRI sensitivity. We first employed a block design stimulus function that consisted of alternating blocks of resting and active conditions. This block was designated as the BHRF. The second HRF was designed by convolving a BHRF with the sum of two gamma functions (from SPM8) and was designated as the CHRF (Friston, 2003; Lindquist et al., 2009). The third one was the impulse response function (IRF) that was fitted to a gamma-variate function [IRF(t) = kt^b^e^-t/c^] appropriate for cerebral blood volume (CBV) weighted fMRI signal under rat whisker stimulation, with *k* = 0.9, *b* = 0.64, and *c* = 4.42 (Lu et al., 2005). The second regressor was the intercept, a vector of ones. A high-pass filter of 1/128 Hz was used to detrend fMRI data (Tanabe et al., 2002).

Three different *t*-maps and magnitude estimate β maps were generated from the corresponding HRF. For different GLM models, the fractional signal change of each voxel was calculated using the same equation as follows:

(1) $$\frac{\Delta \text{S}}{{\text{S}}_{\text{base}}}=\frac{{\text{S}}_{\text{act}}-{\text{S}}_{\text{base}}}{{\text{S}}_{\text{base}}}\times 100=\frac{\beta_{1}}{\beta_{2}}\times 100$$

where S_base_ and S_act_ are the signals at baseline and activation, respectively. β_1_ and β_2_ are the estimated parameters for the two regressors from the GLM (Miao et al., 2014). The resulting *t*- value map and signal-change map were used for the following analysis. Voxels with *t*-values higher than the threshold of 4.8 (corresponding to *P* = 10^-6^) and only groups of at least four activated pixels (Keilholz et al., 2006; Weber et al., 2006) were regarded as significantly activated.

Voxels with *t*-values greater than 4.8 from three HRF analyses were considered as activated voxels and used for regions of interest analysis. Averaged *t*-values and signal changes within S1BF and S1FL were calculated by averaging the *t*-values and signal changes of its constituent activated voxels as defined above, respectively. Differences in *t*-values and estimated BOLD signal changes in the primary somatosensory cortex (S1) among the three HRFs were tested through one-way analysis of variance (ANOVA) tests with repeated measures. If the effect was observed in the ANOVA test results, *post hoc* Tukey’s honest significant difference test was employed. Task-based BOLD data were analyzed using in-house Matlab (The MathWorks, Natick, MA, United States) scripts. Data were expressed as mean ± standard error.

## Results

**FIGURE 1:**
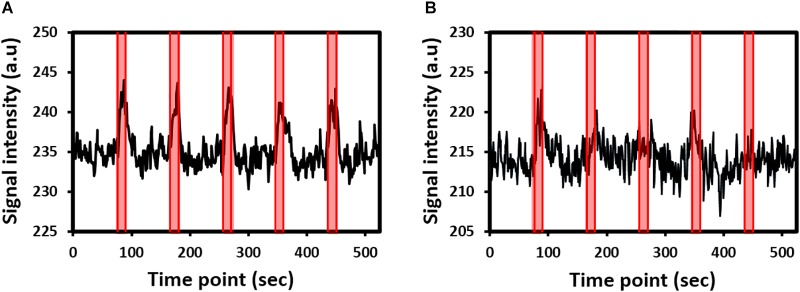
Functional magnetic resonance imaging (fMRI) signal time curves from the primary somatosensory cortex of two representative rats under **(A)** whisker pad stimulation or **(B)** forepaw stimulation. The shaded regions indicate the 15-s stimulation period.

Robust fMRI activations in the contralateral side of the brain were detected in all rats under whisker pad or forepaw stimulation. fMRI signal time curves from the S1 of two representative rats under whisker pad stimulation or forepaw stimulation are shown in Figure 1A,B, respectively. The fMRI signals gradually increased and then gradually decreased to the baseline.

**FIGURE 2:**
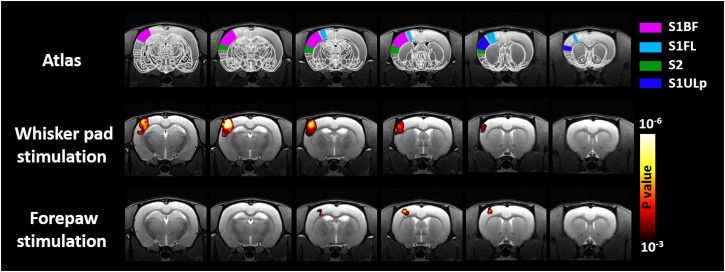
Group-level result of brain regions activated by mysticial pad electrical stimulation and forepaw electrical stimulation. Analyses were performed using a one-sample Student’s *t*-test with a cluster size of four voxels.

Figure 2 shows the group-level activation maps of 10 rats under electric stimulation. The maps shown in this figure were obtained through GLM with CHRF. Consistent with previous reports, robust activations were detected in the S1BF, secondary somatosensory cortex (S2), and primary somatosensory cortex upper lip region (S1ULp) of rats under whisker pad stimulation (Yu et al., 2010). Significant activations in the S1BF associated with forepaw stimulation were found. In line with previous studies (Silva et al., 1999; Shih et al., 2013, 2014), activation in the S2 was not easily detectable in animals under forepaw stimulation. Notably, the volume of the active region in rats under forepaw stimulation was smaller than that in rats under whisker pad stimulation as indicated by the decreased cortical somatosensory representation in the rat brain. The presented activations under whisker pad and forepaw stimulation were similar when employing GLM with the BHRF or IRF was employed.

**FIGURE 3:**
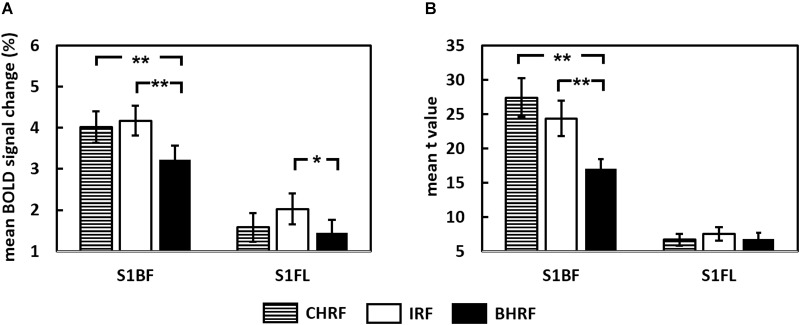
Comparisons of **(A)** estimated BOLD signal changes and **(B)** *t*-statistics between two hemodynamic response functions (HRFs) (^∗∗^*P* < 0.01, ^∗^*P* < 0.05).

Blood-oxygen-level dependent signal changes and *t*-values quantified through GLM analysis are plotted in Figure 3A,B, respectively. The estimated BOLD percentage changes in the S1BF were 4.02 ± 0.38, 4.31 ± 0.42, and 3.22 ± 0.34% when the GLM used CHRF, IRF, and BHRF, respectively. One-way ANOVA with repeated measures showed that HRF had a significant effect on BOLD signal change estimation (*P* < 0.001), where the estimated BOLD signal change in S1BF for GLM with BHRF was significantly lower than those with CHRF (*P* < 0.01) and IRF (*P* < 0.01).

The selection of HRFs also had significant effects on the estimated BOLD signal changes in response to forepaw stimulation (*P* < 0.01). The estimated BOLD signal change in the S1FL for GLM with IRF (1.71 ± 0.34%) was significantly higher than that with BHRF (1.44 ± 0.31%, *P* < 0.05). The comparisons between CHRF and IRF and between CHRF and BHRF were not significantly different (both *P* > 0.05).

The analysis of the influence of HRF selections yielded a similar pattern for the quantification of *t*-values. For whisker pad stimulation, the statistical power of the *t*-values derived from GLM with CHRF or IRF significantly improved relative to those of the *t*-values derived from BHRF (Figure 3B, *P* < 0.001), suggesting that the use of CHRF or IRF improved the statistical significance of voxels. The comparison between CHRF and IRF showed insignificant differences (*P* > 0.05). No significant differences in the quantified *t*-values were detected for the data obtained under forepaw stimulation (*P* = 0.13) when the GLM used any HRF.

**FIGURE 4:**
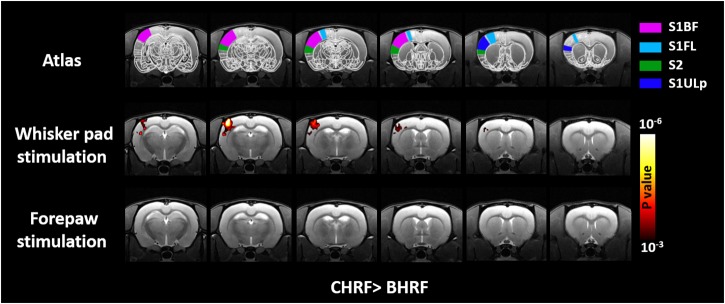
Brain regions showing significant differences in *t*-value quantifications between two HRFs. Relative to that with BHR, GLM with CHRF significantly improved the *t*-statistics for S1BF under whisker pad stimulation. No difference was detected in the opposite direction or under forepaw stimulation in either direction. Analyses were performed using paired Student’s *t*-test with a cluster size of four voxels.

The voxel-wise comparisons of *t*-values between GLM with CHRF and BHRF are displayed in Figure 4. Compared with BHRF, GLM with CHRF significantly improved the activation maps mainly in S1BF under whisker pad stimulation. This result indicated that the CHRF is more appropriate for stimulations with large BOLD signal change. By contrast, BHRF did not increase sensitivity relative to CHRF. No difference was detected under forepaw stimulation in either direction. The comparisons between GLM with IRF and BHRF showed similar patterns.

## Discussion

In this study, we found that the choice of the HRF is crucial in the computation of activations in rat fMRI studies, especially in studies involving stimulations with large BOLD signal changes, such as whisker pad stimulation. We tested three types of HRFs: CHRF, IRF, and BHRF. BHRF is simple and popular among rat fMRI studies (Keilholz et al., 2004; Pelled et al., 2007; Nasrallah et al., 2015). However, its statistical estimations tend to vary with the type of responses. Experimental fMRI data have been used to demonstrate that BHRF affects the results of statistical analyses by underestimating BOLD magnitude changes and *t*-values.

Our results demonstrate that BHRF significantly underestimates signal changes and *t*-statistics. Given that the ground truth is unknown, CHRF or IRF may also be overestimating signal changes and *t*-statistics. In addition, the effect of HRF selection may be dependent on the brain area. To address this issue, we simulated the fMRI signal time curves (Shan et al., 2014) with ground truth HRFs (Supplementary Figure 1) to test the performance of the different HRF models. Numerical simulations showed that for the fMRI signal time curve simulated from the BHRF with BOLD signal changes of more than 1.7%, GLM with CHRF produced *t*-values that were significantly lower than those produced by GLM with BHRF (Supplementary Figure 2A, *P* < 0.05). The same scenario occurred when the fMRI signal time curve was simulated with CHRF but using BHRF in the GLM (Supplementary Figure 2B, *P* < 0.05). The potential explanation to this phenomenon is that when the BOLD signal change is small and the signal-to-noise ratio is low, everything is buried with noise and no difference could be detected among the HRFs, thereby reducing the relative advantage of CHRF. When the BOLD signal change is larger and accompanied with a clear peak instead of a plateau, CHRF is preferred over BHRF due to its existing peak. These simulation results may provide further evidence for the possible underestimation of rodent fMRI results by BHRF, particularly when the BOLD signal change is large and has a clear peak. Moreover, the effect of HRF selection may be independent of the brain area.

Early work done by Chavarrias et al. (2016) showed that a simple approach using a boxcar response provides better model fitting results than complex approaches. This conclusion is not congruent with our present findings. We found that CHRF and IRF improve the statistical power, especially for stimulations with large BOLD signal changes. This deviation may be attributed to the low temporal resolution of 3 s employed in the aforementioned study compared with the 1 s that we used in functional volume acquisition. When temporal resolution is low, the intrinsic hemodynamic response could be blurred and could also cloud the true response, yielding biased estimations (Kim et al., 1997). Additional comprehensive experiments with different temporal resolution settings may help to further parse out this issue.

Our present results provide some interesting insights into the HRF selection in rat fMRI studies. Our data suggest that when the BOLD signal change is large, such as that under whisker pad stimulation, CHRF and IRF are appropriate candidates for modeling the BOLD response, even though IRF is derived from CBV-fMRI, which may provide a response different from that provided by BOLD. The CHRF is derived from the sum of two gamma functions, whereas the IRF is derived from a single gamma function. The results of these HRFs are similar and comparable. This finding is in agreement with that of a previous human study showing that two gamma functions are neither better nor worse than a single gamma function (Handwerker et al., 2004). In CHRF, the second gamma function is included to model the post-stimulus undershoot. We carefully inspected our data and found that the post-stimulus undershoot is not observed in the data for every rat. Thus, inter-subject variation may restrict the statistical power of the approach and may imply that as long as a peak exists instead of a plateau in HRF, the BOLD response can be correctly modeled. It should also be noted that the parameters of the two gamma functions used in this study were empirically derived from SPM. The application of the default setting from SPM to rodent studies may not be appropriate since the parameters in SPM were originally designed for human studies. However, determining the hemodynamic parameters for animal fMRI studies is non-trivial (Silva et al., 2007) and may not be generally performed across studies. In addition, other factors such as the anesthesia regime and the different targeted brain regions may contribute to the variations in hemodynamic parameters. Therefore, the use of the default setting of the two gamma functions from SPM is simple and convenient, thus gaining increasing popularity in rodent fMRI studies (Just et al., 2013; Niranjan et al., 2016). Although the parameters from CHRF were not optimized in this study, the advantage of CHRF over BHRF in improving the statistical power was demonstrated in this work. Additional research similar to the current one but with different hemodynamic parameters can be an important area for future work.

In the field of rodent fMRI studies, Student’s *t*-test (Tenney et al., 2004; Weber et al., 2006; Masamoto et al., 2007; Sanganahalli et al., 2013; Poplawsky and Kim, 2014) and cross-correlation (CC) analysis (Keilholz et al., 2004; Pelled et al., 2007; Shih et al., 2009, 2011; Yang et al., 2013; Nasrallah et al., 2015) are popular statistical strategies for localizing brain regions activated by a task. The principle of Student’s *t*-test is to compare the data between “baseline (off)” and “stimulus (on)” phases, thus providing high *t*-scores for large differences with small standard deviations, and low *t*-scores for small differences with large standard deviations. Notably, the comparison between “on” and “off” corresponds to BHRF and may imply that similar to BHRF, the Student’s *t*-test may underestimate the *t*-value when the BOLD response is large. CC analysis takes a HRF of expected neural responses and correlates it with the MRI signal variations of each voxel. Correlation coefficients are calculated and converted to *t*-values (Hinkle et al., 2003) to generate the activation map. In this regard, the CHRF or IRF can be considered as complementary HRF when employing CC analysis to assess functional activities.

The results in the present work should be interpreted in the context of several limitations. First, in the present study, each rat was subjected to either whisker pad or forepaw electrical stimulation. Therefore, we were unable to make within-rat comparisons. The HRF effect on whisker pad or forepaw stimulations may be affected by physiological differences across rats. The duration of ISO anesthesia is known to influence the functional connectivity in rats (Magnuson et al., 2014). Nevertheless, the time-dependent effects of ISO on electric stimulation fMRI studies remain unclear. As a result, we used separate groups to maintain the same duration of anesthesia in our experiments. The different periods of anesthetization should not be a major concern in the experimental design. Second, electrical stimulation parameters are often dependent on the type of anesthetic (Huttunen et al., 2008; Liu et al., 2013) and sensory system used (Melzer et al., 2006; Just et al., 2010). In this study, the stimulation parameters were 3 Hz and 330 μs electrical pulses. These parameters were first identified to induce robust BOLD response to rat forepaw somatosensory stimulus under alpha-chloralose (Brinker et al., 1999; Silva et al., 2007). Thus, optimal stimulus parameters must be employed to clarify the effects of HRF selection on the analysis of fMRI data obtained through whisker pad stimulation under ISO anesthesia.

## Conclusion

We demonstrated that rat fMRI results could be influenced by HRF selection, especially for stimulations with large BOLD response. BHRF is a simple and straightforward HRF but may underestimate the magnitude of BOLD response and the *t*-values of statistical tests. Sophisticated HRFs, such as CHRF and IRF, provide robust estimation. Our results suggest that CHRF and IRF could serve as complementary HRFs in the analysis of rat fMRI data.

## Author Contributions

S-LP conceptualized the study. S-LP and C-MC designed the experiments and prepared the manuscript. S-CC, C-YH, and W-CS performed the experiments and conducted data analysis. C-TS, C-WH, and S-CC participated in the data collection.

## Conflict of Interest Statement

The authors declare that the research was conducted in the absence of any commercial or financial relationships that could be construed as a potential conflict of interest.

## References

[B1] Aguirre G. K. Zarahn E. D’esposito M. (1998). The variability of human, BOLD hemodynamic responses. *Neuroimage* 8 360–369. 10.1006/nimg.1998.0369 9811554

[B2] Brinker G. Bock C. Busch E. Krep H. Hossmann K. A. Hoehn-Berlage M. (1999). Simultaneous recording of evoked potentials and T2^∗^-weighted MR images during somatosensory stimulation of rat. *Magn. Reson. Med.* 41 469–473. 10.1002/(sici)1522-2594(199903)41:3<469::aid-mrm7>3.3.co;2-0 10204868

[B3] Chavarrias C. Garcia-Vazquez V. Aleman-Gomez Y. Montesinos P. Pascau J. Desco M. (2016). fMRat: an extension of SPM for a fully automatic analysis of rodent brain functional magnetic resonance series. *Med. Biol. Eng. Comput.* 54 743–752. 10.1007/s11517-015-1365-9 26285671

[B4] Friston K. J. (2003). *Statistical Parametric Mapping.* Berlin: Springer.

[B5] Golestani A. M. Wei L. L. Chen J. J. (2016). Quantitative mapping of cerebrovascular reactivity using resting-state BOLD fMRI: validation in healthy adults. *Neuroimage* 138 147–163. 10.1016/j.neuroimage.2016.05.025 27177763 PMC5148619

[B6] Handwerker D. A. Ollinger J. M. D’esposito M. (2004). Variation of BOLD hemodynamic responses across subjects and brain regions and their effects on statistical analyses. *Neuroimage* 21 1639–1651. 10.1016/j.neuroimage.2003.11.029 15050587

[B7] Hinkle D. E. Wiersma W. Jurs S. G. (2003). *Applied Statistics for the Behavioral Sciences*, 5th Edn. Boston: Houghton Mifflin Co.

[B8] Huttunen J. K. Grohn O. Penttonen M. (2008). Coupling between simultaneously recorded BOLD response and neuronal activity in the rat somatosensory cortex. *Neuroimage* 39 775–785. 10.1016/j.neuroimage.2007.06.042 17964186

[B9] Just N. Petersen C. Gruetter R. (2010). BOLD responses to trigeminal nerve stimulation. *Magn. Reson. Imaging* 28 1143–1151. 10.1016/j.mri.2010.02.002 20399585

[B10] Just N. Xin L. Frenkel H. Gruetter R. (2013). Characterization of sustained BOLD activation in the rat barrel cortex and neurochemical consequences. *Neuroimage* 74 343–351. 10.1016/j.neuroimage.2013.02.042 23473934

[B11] Keilholz S. D. Silva A. C. Raman M. Merkle H. Koretsky A. P. (2004). Functional MRI of the rodent somatosensory pathway using multislice echo planar imaging. *Magn. Reson. Med.* 52 89–99. 10.1002/mrm.20114 15236371

[B12] Keilholz S. D. Silva A. C. Raman M. Merkle H. Koretsky A. P. (2006). BOLD and CBV-weighted functional magnetic resonance imaging of the rat somatosensory system. *Magn. Reson. Med.* 55 316–324. 10.1002/mrm.20744 16372281

[B13] Kim S. G. Richter W. Ugurbil K. (1997). Limitations of temporal resolution in functional MRI. *Magn. Reson. Med.* 37 631–636. 10.1002/mrm.1910370427 9094089

[B14] Kim Y. R. Van Meer M. P. Mandeville J. B. Tejima E. Dai G. Topalkara K. (2007). fMRI of delayed albumin treatment during stroke recovery in rats: implication for fast neuronal habituation in recovering brains. *J. Cereb. Blood Flow Metab.* 27 142–153. 10.1038/sj.jcbfm.9600317 16736052

[B15] Lindquist M. A. Meng Loh J. Atlas L. Y. Wager T. D. (2009). Modeling the hemodynamic response function in fMRI: efficiency, bias and mis-modeling. *Neuroimage* 45 S187–S198. 10.1016/j.neuroimage.2008.10.065 19084070 PMC3318970

[B16] Liu J. V. Hirano Y. Nascimento G. C. Stefanovic B. Leopold D. A. Silva A. C. (2013). fMRI in the awake marmoset: somatosensory-evoked responses, functional connectivity, and comparison with propofol anesthesia. *Neuroimage* 78 186–195. 10.1016/j.neuroimage.2013.03.038 23571417 PMC3778909

[B17] Liu Z. M. Schmidt K. F. Sicard K. M. Duong T. Q. (2004). Imaging oxygen consumption in forepaw somatosensory stimulation in rats under isoflurane anesthesia. *Magn. Reson. Med.* 52 277–285. 10.1002/mrm.20148 15282809 PMC2962950

[B18] Lu H. Soltysik D. A. Ward B. D. Hyde J. S. (2005). Temporal evolution of the CBV-fMRI signal to rat whisker stimulation of variable duration and intensity: a linearity analysis. *Neuroimage* 26 432–440. 10.1016/j.neuroimage.2005.02.016 15907301

[B19] Magnuson M. E. Thompson G. J. Pan W. J. Keilholz S. D. (2014). Time-dependent effects of isoflurane and dexmedetomidine on functional connectivity, spectral characteristics, and spatial distribution of spontaneous BOLD fluctuations. *NMR Biomed.* 27 291–303. 10.1002/nbm.3062 24449532 PMC4465547

[B20] Masamoto K. Kim T. Fukuda M. Wang P. Kim S. G. (2007). Relationship between neural, vascular, and BOLD signals in isoflurane-anesthetized rat somatosensory cortex. *Cereb. Cortex* 17 942–950. 10.1093/cercor/bhl005 16731882

[B21] Melzer P. Champney G. C. Maguire M. J. Ebner F. F. (2006). Rate code and temporal code for frequency of whisker stimulation in rat primary and secondary somatic sensory cortex. *Exp. Brain Res.* 172 370–386. 10.1007/s00221-005-0334-1 16456683

[B22] Miao X. Gu H. Yan L. Lu H. Wang D. J. Zhou X. J. (2014). Detecting resting-state brain activity by spontaneous cerebral blood volume fluctuations using whole brain vascular space occupancy imaging. *Neuroimage* 84 575–584. 10.1016/j.neuroimage.2013.09.019 24055705

[B23] Nasrallah F. A. Yeow L. Y. Biswal B. Chuang K. H. (2015). Dependence of BOLD signal fluctuation on arterial blood CO2 and O2: Implication for resting-state functional connectivity. *Neuroimage* 117 29–39. 10.1016/j.neuroimage.2015.05.035 26003858

[B24] Niranjan A. Christie I. N. Solomon S. G. Wells J. A. Lythgoe M. F. (2016). fMRI mapping of the visual system in the mouse brain with interleaved snapshot GE-EPI. *Neuroimage* 139 337–345. 10.1016/j.neuroimage.2016.06.015 27296012 PMC4988789

[B25] Ogawa S. Lee T. M. Kay A. R. Tank D. W. (1990). Brain magnetic resonance imaging with contrast dependent on blood oxygenation. *Proc. Natl. Acad. Sci. U.S.A.* 87 9868–9872. 2124706 10.1073/pnas.87.24.9868 PMC55275

[B26] Ogawa S. Tank D. W. Menon R. Ellermann J. M. Kim S. G. Merkle H. (1992). Intrinsic signal changes accompanying sensory stimulation: functional brain mapping with magnetic resonance imaging. *Proc. Natl. Acad. Sci. U.S.A.* 89 5951–5955. 10.1073/pnas.89.13.5951 1631079 PMC402116

[B27] Pelled G. Chuang K. H. Dodd S. J. Koretsky A. P. (2007). Functional MRI detection of bilateral cortical reorganization in the rodent brain following peripheral nerve deafferentation. *Neuroimage* 37 262–273. 10.1016/j.neuroimage.2007.03.069 17544301 PMC2253720

[B28] Poplawsky A. J. Kim S. G. (2014). Layer-dependent BOLD and CBV-weighted fMRI responses in the rat olfactory bulb. *Neuroimage* 91 237–251. 10.1016/j.neuroimage.2013.12.067 24418506 PMC3965612

[B29] Ramos-Cabrer P. Justicia C. Wiedermann D. Hoehn M. (2010). Stem cell mediation of functional recovery after stroke in the rat. *PLoS One* 5:e12779. 10.1371/journal.pone.0012779 20877642 PMC2943902

[B30] Rana M. Gupta N. Dalboni Da Rocha J. L. Lee S. Sitaram R. (2013). A toolbox for real-time subject-independent and subject-dependent classification of brain states from fMRI signals. *Front. Neurosci.* 7:170. 10.3389/fnins.2013.00170 24151454 PMC3798026

[B31] Sanganahalli B. G. Herman P. Behar K. L. Blumenfeld H. Rothman D. L. Hyder F. (2013). Functional MRI and neural responses in a rat model of Alzheimer’s disease. *Neuroimage* 79 404–411. 10.1016/j.neuroimage.2013.04.099 23648961 PMC3700380

[B32] Shan Z. Y. Wright M. J. Thompson P. M. Mcmahon K. L. Blokland G. G. De Zubicaray G. I. (2014). Modeling of the hemodynamic responses in block design fMRI studies. *J. Cereb. Blood Flow Metab.* 34 316–324. 10.1038/jcbfm.2013.200 24252847 PMC3915209

[B33] Shih Y. Y. Chen C. C. Shyu B. C. Lin Z. J. Chiang Y. C. Jaw F. S. (2009). A new scenario for negative functional magnetic resonance imaging signals: endogenous neurotransmission. *J. Neurosci.* 29 3036–3044. 10.1523/JNEUROSCI.3447-08.2009 19279240 PMC6666445

[B34] Shih Y. Y. Chen Y. Y. Lai H. Y. Kao Y. C. Shyu B. C. Duong T. Q. (2013). Ultra high-resolution fMRI and electrophysiology of the rat primary somatosensory cortex. *Neuroimage* 73 113–120. 10.1016/j.neuroimage.2013.01.062 23384528 PMC3733488

[B35] Shih Y. Y. Huang S. Chen Y. Y. Lai H. Y. Kao Y. C. Du F. (2014). Imaging neurovascular function and functional recovery after stroke in the rat striatum using forepaw stimulation. *J. Cereb. Blood Flow Metab.* 34 1483–1492. 10.1038/jcbfm.2014.103 24917039 PMC4158660

[B36] Shih Y. Y. Wey H. Y. De La Garza B. H. Duong T. Q. (2011). Striatal and cortical BOLD, blood flow, blood volume, oxygen consumption, and glucose consumption changes in noxious forepaw electrical stimulation. *J. Cereb. Blood Flow Metab.* 31 832–841. 10.1038/jcbfm.2010.173 20940730 PMC3063626

[B37] Silva A. C. Koretsky A. P. Duyn J. H. (2007). Functional MRI impulse response for BOLD and CBV contrast in rat somatosensory cortex. *Magn. Reson. Med.* 57 1110–1118. 10.1002/mrm.21246 17534912 PMC4756432

[B38] Silva A. C. Lee S. P. Yang G. Iadecola C. Kim S. G. (1999). Simultaneous blood oxygenation level-dependent and cerebral blood flow functional magnetic resonance imaging during forepaw stimulation in the rat. *J. Cereb. Blood Flow Metab.* 19 871–879. 10.1097/00004647-199908000-00006 10458594

[B39] Tanabe J. Miller D. Tregellas J. Freedman R. Meyer F. G. (2002). Comparison of detrending methods for optimal fMRI preprocessing. *Neuroimage* 15 902–907. 10.1006/nimg.2002.1053 11906230

[B40] Tenney J. R. Duong T. Q. King J. A. Ferris C. F. (2004). FMRI of brain activation in a genetic rat model of absence seizures. *Epilepsia* 45 576–582. 10.1111/j.0013-9580.2004.39303.x 15144421 PMC2949946

[B41] Tsurugizawa T. Uematsu A. Uneyama H. Torii K. (2010). Effects of isoflurane and alpha-chloralose anesthesia on BOLD fMRI responses to ingested L-glutamate in rats. *Neuroscience* 165 244–251. 10.1016/j.neuroscience.2009.10.006 19819307

[B42] Weber R. Ramos-Cabrer P. Wiedermann D. Van Camp N. Hoehn M. (2006). A fully noninvasive and robust experimental protocol for longitudinal fMRI studies in the rat. *Neuroimage* 29 1303–1310. 10.1016/j.neuroimage.2005.08.028 16223588

[B43] Wu W. C. Lien S. H. Chang J. H. Yang S. C. (2014). Caffeine alters resting-state functional connectivity measured by blood oxygenation level-dependent MRI. *NMR Biomed.* 27 444–452. 10.1002/nbm.3080 24478235 PMC4260672

[B44] Yang P. F. Chen Y. Y. Chen D. Y. Hu J. W. Chen J. H. Yen C. T. (2013). Comparison of fMRI BOLD response patterns by electrical stimulation of the ventroposterior complex and medial thalamus of the rat. *PLoS One* 8:e66821. 10.1371/journal.pone.0066821 23826146 PMC3691267

[B45] Yu X. Glen D. Wang S. Dodd S. Hirano Y. Saad Z. (2012). Direct imaging of macrovascular and microvascular contributions to BOLD fMRI in layers IV-V of the rat whisker-barrel cortex. *Neuroimage* 59 1451–1460. 10.1016/j.neuroimage.2011.08.001 21851857 PMC3230765

[B46] Yu X. Wang S. Chen D. Y. Dodd S. Goloshevsky A. Koretsky A. P. (2010). 3D mapping of somatotopic reorganization with small animal functional MRI. *Neuroimage* 49 1667–1676. 10.1016/j.neuroimage.2009.09.021 19770051 PMC2967485

