# Supplementary material for: Effects of Hemodynamic Response Function Selection on Rat fMRI Statistical Analyses
Source: Front Neurosci. 2019 Apr 30;13:400. doi: 10.3389/fnins.2019.00400 (PMC6503084; doi:10.3389/fnins.2019.00400)
Supplement: Supplementary file 1 [file Image_1.pdf]

Effects of hemodynamic response function selection on rat fMRI statistical analyses

Shin-Lei Peng,[1*†] Chun-Ming Chen,[2†] Chen-You Huang,[1] Cheng-Ting Shih,[3] Chiun-Wei Huang,[4] Shao-Chieh Chiu,[4] and Wu-Chung Shen,[1,2]

[1]Department of Biomedical Imaging and Radiological Science, China Medical University, Taichung, Taiwan; [2]Department of Radiology, China Medical University Hospital, Taichung, Taiwan; [3] Department of Medical Imaging and Radiological Sciences, Chung Shan Medical University, Taichung, Taiwan; [4]Center for Advanced Molecular Imaging and Translation, Chang Gung Memorial Hospital, Taoyuan, Taiwan;

[†] The first two authors (Shin-Lei Peng and Chun-Ming Chen) contributed equally to this work.

Corresponding Author
Shin-Lei Peng, PhD
Department of Biomedical Imaging and Radiological Science
China Medical University, Taichung, Taiwan
91 Hsueh-Shih Road, Taichung, Taiwan, 40402
E-mail: speng@mail.cmu.edu.tw
Tel: +886-4-22053366-7710
Fax: +886-4-22081447

Supplementary Materials

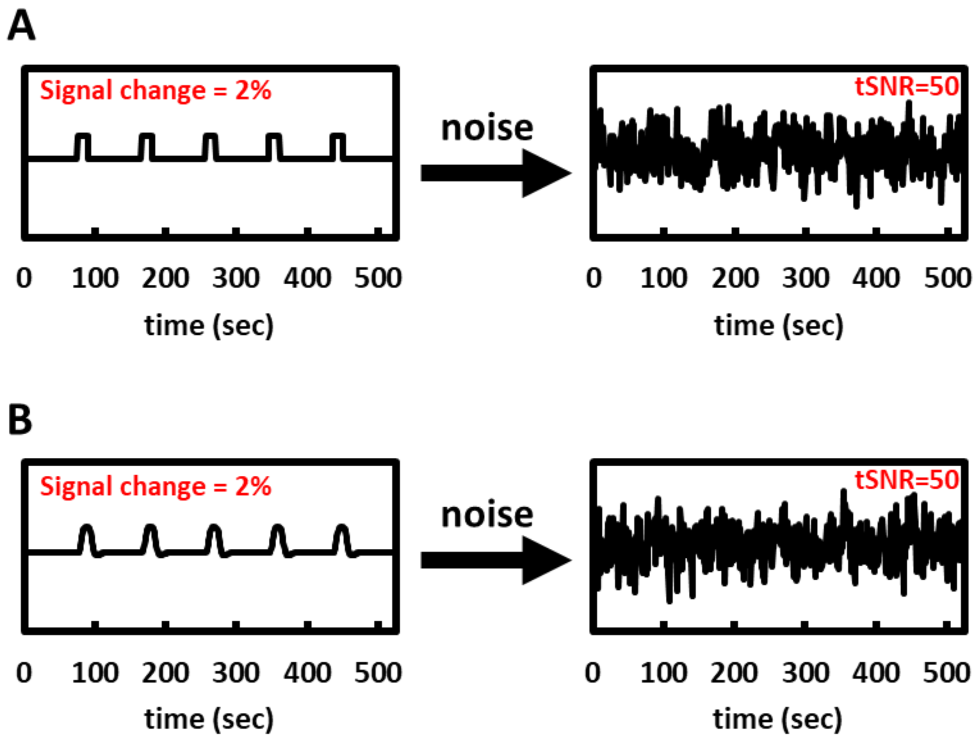

Fig. 1 Procedures for fMRI signal curve simulations. The top row (A) shows the fMRI signal time curves simulated from BHRF. The bottom row (B) shows the fMRI signal time curves simulated from CHRF. tSNR, temporal signal-to-noise ratio.

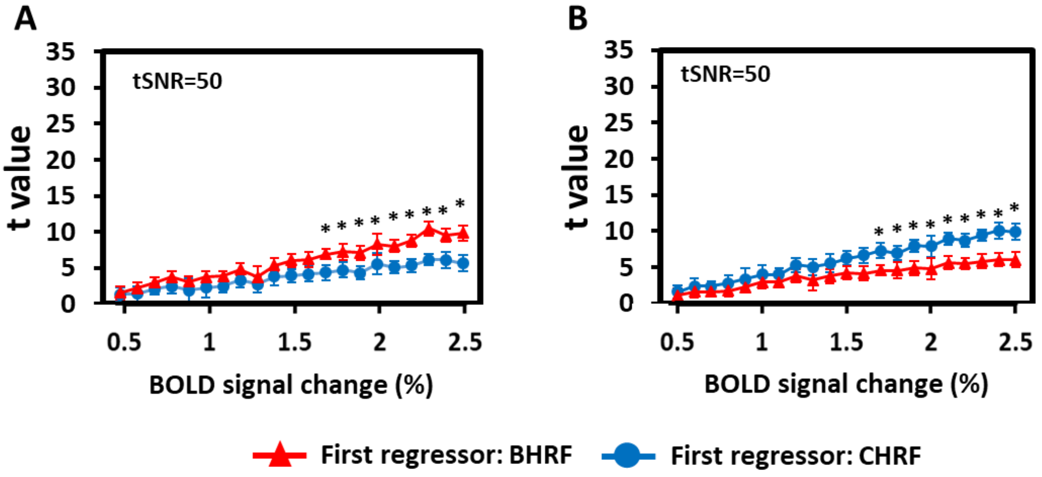

Fig. 2 Comparisons of t-values derived from the general linear model with either BHRF or CHRF as the first regressor in the simulation study. (A) *t*-values for the fMRI signal time curve simulated through BHRF; (B) *t*-values for the fMRI signal time curve simulated through CHRF. tSNR, temporal signal-to-noise ratio. *: P < 0.002.
